# Supplementary figures and images for: The mammalian rhomboid protein RHBDL4 protects against endoplasmic reticulum stress by regulating the morphology and distribution of ER sheets
Source: J Biol Chem. 2022 Apr 15;298(6):101935. doi: 10.1016/j.jbc.2022.101935 (PMC9136127; doi:10.1016/j.jbc.2022.101935)

Figure S1 HeLa

HA-RHBDL4 WT

RHBDL3-KDEL

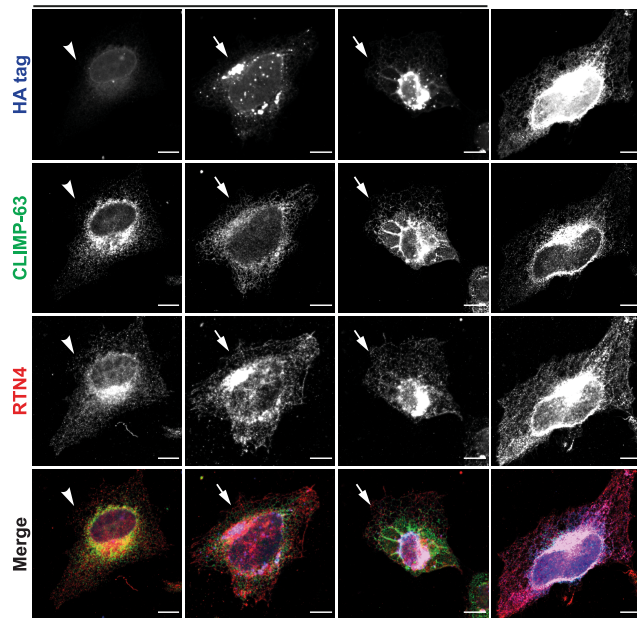

Supplement: Supplemental Figure S1 [file mmc1.pdf]

Figure S2

U2OS

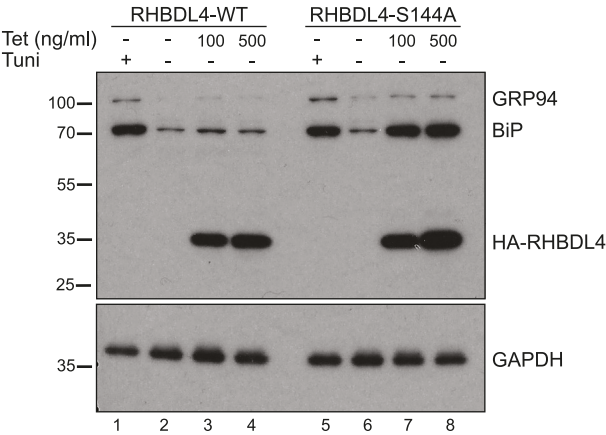

Supplement: Supplemental Figure S2 [file mmc2.pdf]

Figure S3

HeLa

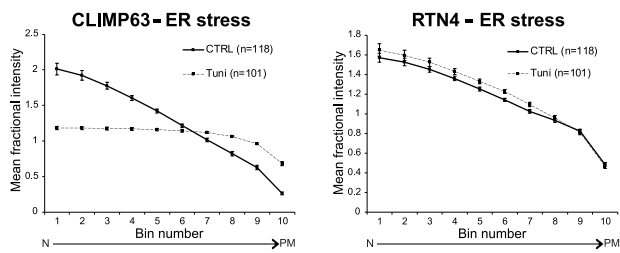

Supplement: Supplemental Figure S3 [file mmc3.pdf]

Figure S4

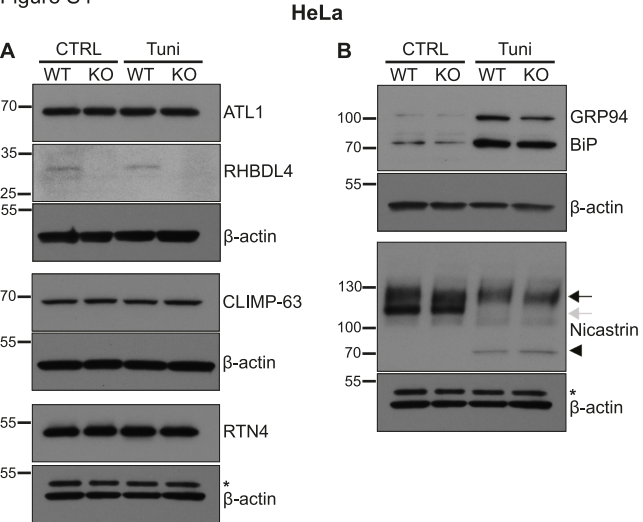

Supplement: Supplemental Figure S4 [file mmc4.pdf]

Figure S5

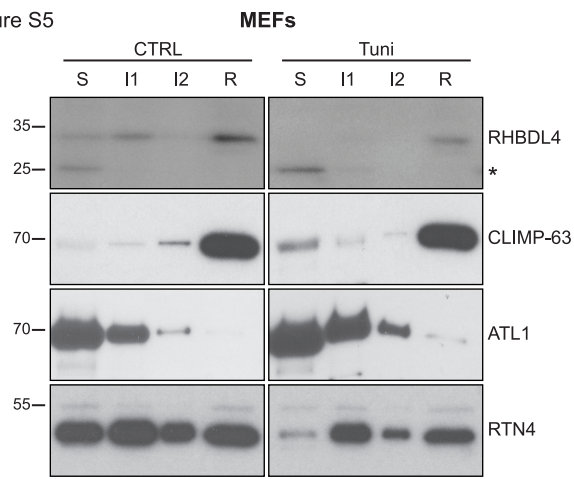

Supplement: Supplemental Figure S5 [file mmc5.pdf]

Figure S6

MEFs

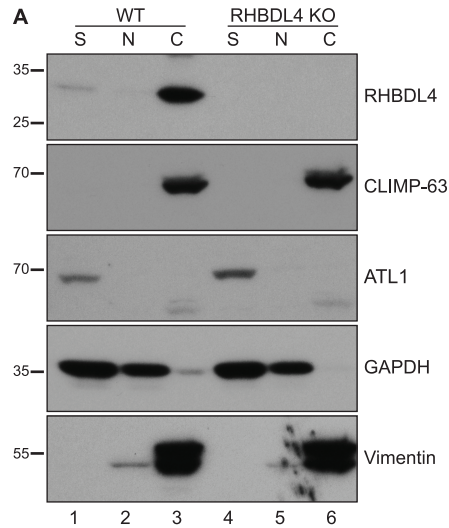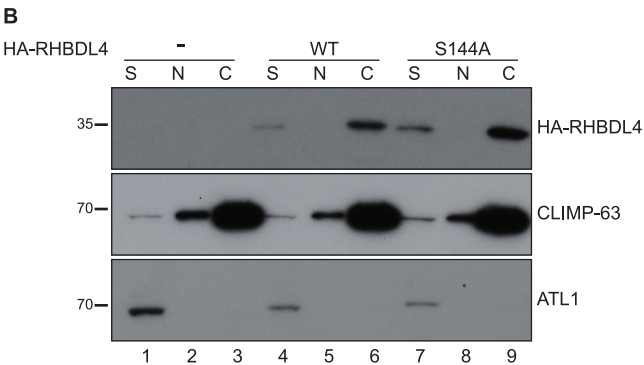

Supplement: Supplemental Figure S6 [file mmc6.pdf]

Figure S7

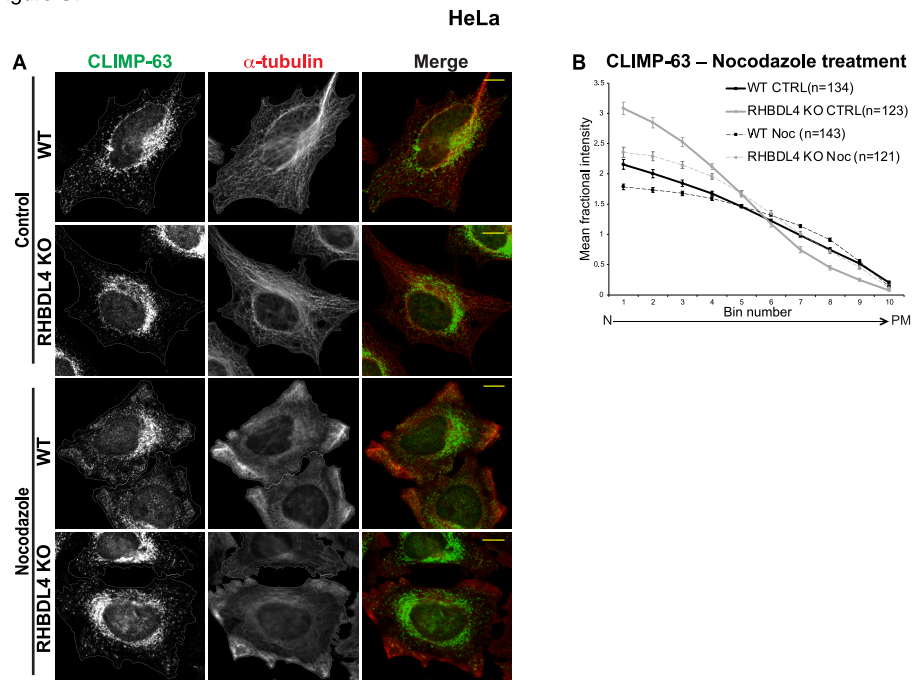

Supplement: Supplemental Figure S7 [file mmc7.pdf]

Figure S8

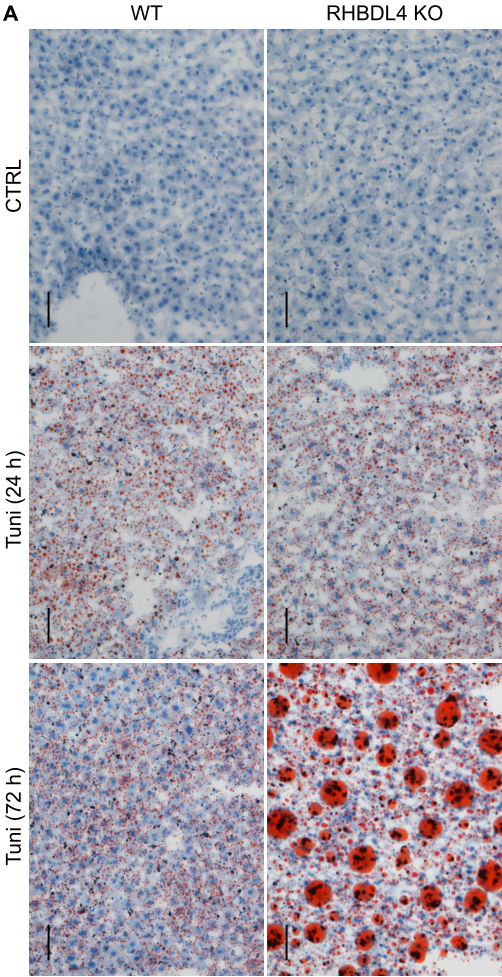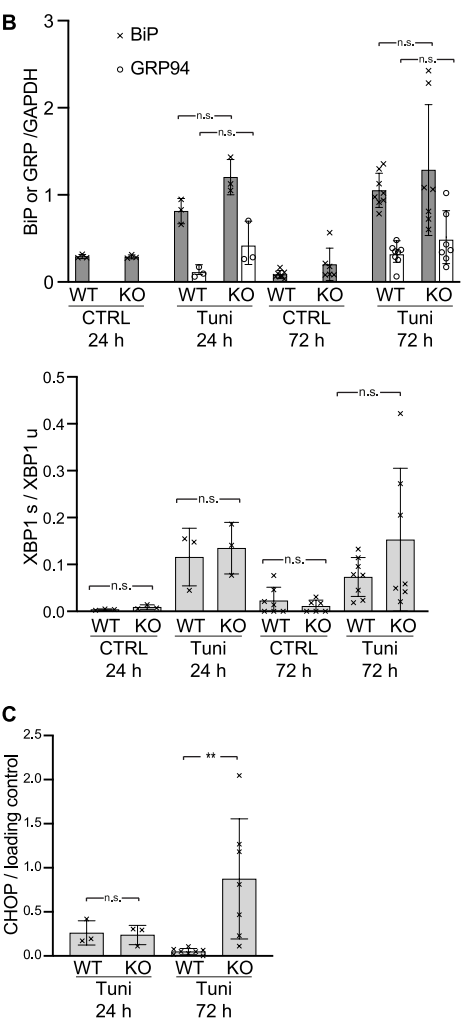

Supplement: Supplemental Figure S8 [file mmc8.pdf]
