# Supplementary material for: The mammalian rhomboid protein RHBDL4 protects against endoplasmic reticulum stress by regulating the morphology and distribution of ER sheets
Source: J Biol Chem. 2022 Apr 15;298(6):101935. doi: 10.1016/j.jbc.2022.101935 (PMC9136127; doi:10.1016/j.jbc.2022.101935)
Supplement: Supplemental Figure S9 [file mmc9.pdf]

RHBDL4 protects against endoplasmic reticulum stress by regulating the morphology and distribution of ER sheets

Viorica L. Lastun, Clémence Levet and Matthew Freeman

Supplementary figure legends

Figure S1. RHBDL4 overexpression disrupts the ER organisation

Immunofluorescence images of RHBDL4 (left) and RHBDL3-KDEL (right) overexpression in HeLa cells. Cells were transfected with HA-tagged RHBDL4 or RHBDL3-KDEL and, after 24 hours, stained for HA tag (blue), CLIMP-63 (green) and RTN4 (red). Scale bar 10 μm. The arrowhead and arrows indicate cells with low and high RHBDL4 expression levels, respectively. The images are representative of three independent experiments.

Figure S2. The expression of catalytically inactive RHBDL4 induces ER stress and triggers the UPR

Western blots of UPR targets BiP and GRP94 (both detected by using an anti-KDEL antibody) in total cell lysates of U2OS Tet-On cells expressing HA-tagged RHBDL4 WT or RHBDL4 S144A. The membrane was incubated with the anti-KDEL and anti-HA antibodies simultaneously. As a positive control for UPR induction, cells were treated with tunicamycin for 24 hours. GAPDH was used as a loading control. The results are representative of three independent experiments.

Figure S3. The distribution of CLIMP-63 and RTN4 in tunicamycin treated HeLa cells

Quantification of CLIMP-63 (left) and RTN4 (right) distribution in HeLa cells control or 24 hours after tunicamycin treatment. Representative images are presented in Fig. 3*A* (control) and 3*F* (24 hours tunicamycin).

Figure S4. The levels of ER shaping proteins and the ER stress response are similar in WT and RHBDL4 KO cells

Western blot of total cell lysates from WT and RHBDL4 KO HeLa in control conditions or 24 hours after tunicamycin treatment. ***A*,** Western blots of RHBDL4 and

ER shaping proteins ATL1, CLIMP-63 and RTN4. ***B,*** Western blots of UPR targets BiP and GRP94 (detected using an anti-KDEL antibody), and nicastrin. The black arrow represents the mature post-Golgi protein, while the grey arrow represents the immature, ER-localised one. The arrowhead represents the non-glycosylated nicastrin, visible in the tunicamycin-treated samples. β-actin was used as a loading control and it was developed on the stripped membranes, previously used for detection of the proteins shown above each β-actin panel. The asterisk represents incompletely removed RTN4. The results are representative of three independent experiments.

Figure S5. RHBDL4 resides within the ER sheets

Microsomes from MEFs, control and tunicamycin treated, were separated as in Figure 5*A*. Smooth ER (S), rough ER (R) and intermediate ER (I) were analysed by western blot for the indicated ER proteins. The asterisk represents a non-specific band. The results are representative of three independent experiments.

Figure S6. RHBDL4 associates with the cytoskeleton

A, Western blot of soluble (S), nuclear (N) and cytoskeletal (C) fractions isolated from WT and RHBDL4 KO MEFs and analysed for ER proteins – RHBDL4, CLIMP-63, ATL1 – as well as for GAPDH and vimentin. ***B***, Anti-HA tag western blot of C, N and S fractions isolated from RHBDL4 KO MEFs transiently expressing HA-tagged RHBDL4 WT or RHBDL4 S144A. The results are representative of three independent experiments (***A***) or two independent experiments (***B***).

Figure S7. RHBDL4 knockout affects the ER-sheet re-distribution when microtubules are depolymerised

A, Immunofluorescence of WT and RHBDL4 KO HeLa cells showing the ER-sheet (CLIMP-63 – green) and microtubule (α-tubulin – red) distribution under control or nocodazole treatment. ***B***, Quantification of CLIMP-63 distribution. Error bars represent the 95% CI. The results are representative of three independent experiments.

Figure S8. RHBDL4 protects against the ER stress in mice

Oil red O staining of liver tissue sections from WT and RHBDL4 KO mice, control, 24 or 72 hours after tunicamycin treatment. The images are representative of one experiment with n=3 mice per genotype for 24 hours treatment and n=2 mice per genotype for 72 hours treatment; n=4 mice per genotype for control. *B.* Quantification of BiP and GRP94 western blots (upper panel) and XBP1 splicing RT-PCR (lower panel) from mouse liver: representative images are shown in Fig. 8*C.* BiP and GRP94 signal was normalised to GAPDH (the loading control). Error bars represent standard deviation. n=3 mice per genotype per condition for 24 hours treatment and n=6 to 8 mice per genotype per condition for 72 hours treatment. *C.* Quantification of CHOP western blots. CHOP signal was normalised to the non-specific band indicated in Fig. 8*C.* Error bars represent standard deviation. p=0.8205 t-test, two-sided, and p=1.00 Mann-Whitney-Wilcoxon test, two-sided, for 24 hours treatment; p=0.0044 t-test, two-sided, (two asterisks on the graph) and p=0.0003 Mann-Whitney-Wilcoxon test, two-sided, for 72 hours treatment.
